# Frontiers in Neurotransmitter Probes: Innovations and Perspectives in Norepinephrine Detection Technology

**DOI:** 10.1155/jamc/5515088

**Published:** 2026-10-17

**Authors:** Rufei Ma, Zhao Wang

**Affiliations:** ^1^ School of Physical Education and International Equestrian Academy Wuhan Business University Wuhan 430056 China wbu.edu.cn; ^2^ Research Center for Modern Equine Industry Development Wuhan Business University Wuhan 430056 China wbu.edu.cn; ^3^ Hubei Provincial Engineering Research Center of Racing Horse Detection and Application Transformation Equine Science Research and Horse Doping Control Laboratory Wuhan Business University Wuhan 430056 China wbu.edu.cn

**Keywords:** excited-state intramolecular proton transfer, ferroptosis, glutathione, near-infrared probe, stroke

## Abstract

Norepinephrine (NE), the primary neurotransmitter of locus coeruleus projections, plays a crucial role in regulating arousal, emotion, memory, and stress responses. Its dysregulation is closely associated with various neurological disorders such as depression, Parkinson’s disease, and epilepsy. However, conventional detection techniques such as microdialysis coupled with liquid chromatography are constrained by limited spatiotemporal resolution and inherent invasiveness, largely due to NE’s low concentration, rapid metabolism, and complex distribution in vivo. These limitations make real‐time, in situ dynamic monitoring particularly challenging. In recent years, fluorescence and bioluminescence probe technologies, founded on molecular recognition or self‐assembly strategies, have enabled notable advances in the in situ detection and imaging of NE, owing to their superior spatiotemporal resolution, favorable biocompatibility, and versatile design. This review summarizes several representative studies in the field, covering probe design principles, response mechanisms, analytical performance, and biological applications. It also highlights emerging frontiers such as subcellular localization, in vivo dynamic imaging, millisecond‐scale monitoring, multitarget cooperative recognition, and integrated diagnosis and therapy. These technologies have deepened our understanding of the spatiotemporal dynamics of NE in physiological and pathological processes, while also providing powerful tools for mechanistic research, early diagnosis, and precise treatment of neuropsychiatric disorders.

## 1. Introduction

Norepinephrine (NE) is synthesized from tyrosine via metabolic pathways and is primarily stored in vesicles within nerve endings and the adrenal medulla, playing extensive neuromodulatory roles in various physiological activities of the human body. The dynamic release of NE in the brain widely participates in and regulates multiple physiological processes. It not only profoundly influences advanced neural functions such as stress response, sleep–wake cycles, attention, and cognitive focus but also modulates neuroimmune interactions and inflammatory responses [1]. Additionally, as an important chemical messenger of the autonomic nervous system, NE plays a critical regulatory role in peripheral cardiovascular activities, visceral functions, and other autonomic nervous responses [2–4]. Therefore, precise detection of NE is indispensable for elucidating its roles in both physiological and pathological processes. A variety of detection methods have been developed for the quantitative analysis of NE, including capillary electrophoresis, mass spectrometry, liquid chromatography, electrochemical detection, flow immunoassay, and native fluorescence detection [5–9]. However, NE presents challenges for in vivo detection due to its low concentration, rapid metabolism (millisecond‐scale release), and complex spatial distribution (spanning cellular organelles to brain regions). Most of these methods rely on the analysis of ex vivo samples, making it difficult to achieve in situ, real‐time monitoring of NE in the nervous system. They are also susceptible to interference from analogs such as dopamine (DA) and epinephrine (EP), amino acids including glutamate (Glu) and *γ*‐aminobutyric acid (GABA), aromatic monoamines such as serotonin and histamine, and acetylcholine (ACh). Each neurotransmitter has distinct roles [10]. DA is associated with motivation, reward, and pleasure [11], while serotonin plays a key role in regulating mood and digestion [12]. NE influences attention and the response to stress [13]. ACh is involved in muscle movement and cognitive abilities [13]. Glu, the primary excitatory neurotransmitter, is essential for cognitive functions such as learning and memory [14]. In contrast, GABA, the main inhibitory neurotransmitter in the brain, helps control fear and anxiety [15].

Moreover, ex vivo NE is prone to oxidative degradation in the external environment, making it difficult to maintain its stability and bioactivity. This limits the reliability and physiological relevance of studies based on such samples for revealing NE dynamic signaling and metabolic pathways. With the development of noninvasive imaging techniques and the continuous innovation of analytical methods, fluorescence labeling technology has provided an important visualization tool for real‐time observation of target molecules in complex biological environments in vivo [16–18]. By designing fluorescent probes with high specificity and biocompatibility, dynamic monitoring of target molecule distribution, concentration changes, and their involvement in physiological activities can be achieved at the tissue or even whole‐organism level. Meanwhile, recognition sites engineered into the probe structure can effectively avoid interference from other neurotransmitters and similar bioactive small molecules [19, 20].

This review systematically summarizes key breakthroughs from over a dozen representative studies on NE detection (Table 1), focusing on probe design principles, response mechanisms, analytical performance, and biological applications, aiming to provide insights for future developments in this field.

**TABLE 1:** Representative NE fluorescent probes.

Probe	Sensing mode	Ex/Em (nm)	Time	Linear range (mM)	Fluorescence enhancement multiples	Application
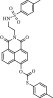	Turn on	450/575	60 min	0–5	—	In cells

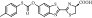	Turn on	—	30 min	0–0.25	41‐fold	In cells In vivo

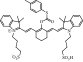	Turn on	550/640	60 min	—	—	In cells In vivo

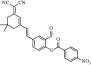	Turn on	440/635	12 min	0–5	—	In cells In vivo

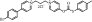	Turn off	360/480	100 ms	0–3 × 10^−4^	—	In cells In vivo

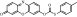	Turn on	570/590	30 min	0.2–1.8	7‐fold	In cells In vivo

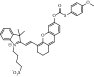	Turn on	585/720	60 ms	1 × 10^−4^–5 × 10^−3^	15‐fold	In cells In vivo

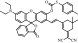	Turn on	543/725	60 min	0–5	—	In cells

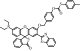	Turn on	365/533	10 min	0–5	38.5‐fold	In cells

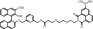	Ratio fluorescence	350/400,526	30 min	0–5 × 10^−3^	—	In cells In vivo

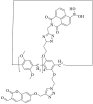	Turn off	435/760	—	1–2.1 × 10^−2^	—	In cells In vivo

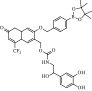	Turn on	375/460	30 min	—	—	In cells In vivo

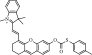	Turn on	650/724	5 h	0–5	—	In cells

### 1.1. Paradigm Shift in Spatiotemporal Neurotransmitter Monitoring

As a core messenger for neural communication, the physiological functions of NE are rooted in its highly dynamic spatiotemporal distribution. At the subcellular scale, transient fluctuations in NE concentration within specific organelle microenvironments (e.g., endoplasmic reticulum [ER], vesicles) directly regulate key intracellular pathways such as calcium signal and protein phosphorylation [21–23]. At the neural network level, the kinetics of NE release, diffusion, and reuptake across different brain regions form the basis for information encoding and transmission within neural circuits. However, traditional detection technologies, limited by inherent invasiveness and fragmented spatiotemporal resolution, have struggled to fully capture this cross‐scale dynamic process.

Recent innovations in molecular probe technology have led to fundamental breakthroughs in observational capabilities. On the one hand, breakthroughs in spatially targeted localization enable probes to navigate precisely to specific organelles or brain regions, allowing for the in situ analysis of NE distribution from organelle microdomains to the whole living brain. On the other hand, the development of ultrafast response mechanisms has elevated temporal resolution to the millisecond scale, enabling for the first time the in situ capture of transient NE signals in contexts such as the synaptic cleft. This synergistic leap in spatial precision and temporal resolution has fundamentally transformed the observational paradigm of neurotransmitter research—shifting from static “snapshot” analysis to continuous tracking of four‐dimensional dynamics (three‐dimensional space + time). This shift provides an indispensable observational window for revealing the real‐time mechanisms of NE in memory encoding, emotion regulation, and disease pathogenesis, powerfully propelling neuroscience into a new era of “dynamic visualization.”

### 1.2. Organelle‐Level Imaging

The team led by Lin Weiying developed an ER‐targeted fluorescent probe [24], ER‐NE, based on a 1,8‐naphthalimide fluorophore conjugated with a *p*‐toluenesulfonamide targeting group, achieving high ER localization efficiency (90%). The probe reacts with NE via a thioester bond, releasing the fluorophore and enabling dynamic monitoring of NE within the ER (response time ∼60 min) (Figure 1). Moreover, near‐physiological pH, the probe ER‐NE exhibits a good response specifically to NE, while showing little to no response to other neurotransmitters and small‐molecule analogs. In PC12 cells, high potassium stimulation induced NE exocytosis within 2 min, reflected by a 48.7% decrease in fluorescence intensity, whereas in a corticosterone‐induced depression model, the NE exocytosis rate was significantly reduced. Colocalization experiments confirmed strong ER enrichment of the probe (Pearson’s coefficient = 0.9065). This work provides the first evidence at the subcellular level linking NE‐mediated ER stress to depression, offering a new perspective on the regulation of organelle function by neurotransmitters.

**FIGURE 1:**
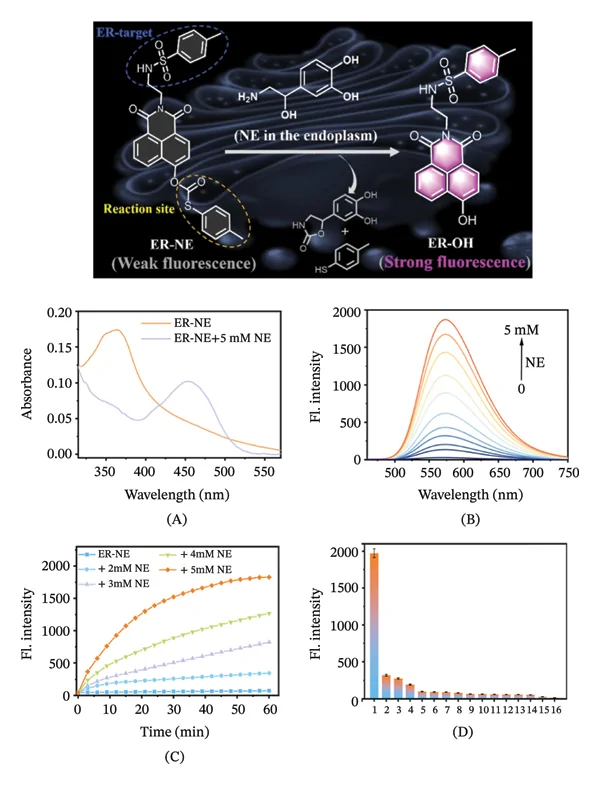
Identification and Colocalization of the Probe ER‐NE [24].

Achieving noninvasive, highly specific dynamic imaging of NE at the live‐animal level is a key objective in neurochemical research. The team led by Feng Ping at Sichuan University developed an NE‐specific probe [25], NBP, based on bioluminescence imaging (BLI) technology (Figure 2). The probe employs an “off‐on” design, with a *p*‐thiolcresol group protecting a luciferase substrate. When NE cleaves the C–S bond via a nucleophilic substitution reaction, the released luciferin activates a bioluminescent signal. The probe demonstrates high sensitivity (detection limit 1 μM, linear range 0–250 μM) and excellent selectivity in vitro (response to analogs such as DA and serotonin, as well as biological matrix interferents, is only 1/15 to 1/45 of that to NE). Its core breakthrough lies in achieving dynamic monitoring in vivo, in luciferase‐expressing cells, NBP visualized concentration‐dependent changes of exogenous NE (0–200 μM), in transgenic FVB‐luc + mice, systemic imaging after intravenous probe injection showed that NE administration (0.2 mM) via the tail vein induced approximately a 2.9‐fold signal enhancement (response time ≤ 18 min). The study further confirmed that after intervention with the antidepressant fluoxetine, NE levels in brain regions increased in a dose‐dependent manner (3.32‐fold signal increase at 100 mg/kg). Leveraging the no‐excitation‐light‐required nature of bioluminescence, this technology effectively avoids tissue autofluorescence interference, realizing for the first time noninvasive real‐time visualization of NE dynamics in living mammals, providing an important tool for studying neurotransmitter regulation and drug effects.

**FIGURE 2:**
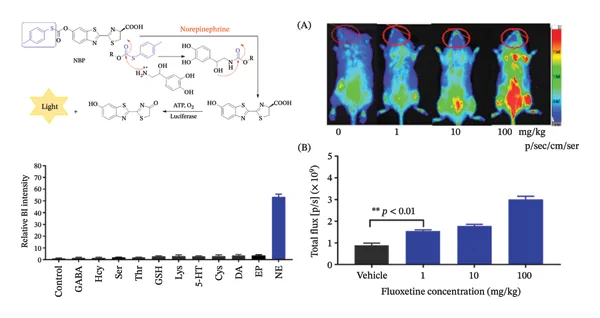
Sensing mechanism of probe NBP [25].

On the other hand, the team of Yin Caixia and Huo Fangjun at Shanxi University developed a novel specific fluorescent probe for highly selective NE detection based on a “protection‐deprotection” strategy, using a modified water‐soluble cyanine dye as the fluorophore (Figure 3). In in vitro tests [26], probe 3 also exhibited good selectivity, effectively avoiding interference from other amine neurotransmitters. The probe 3 was successfully applied at multiple levels; in PC12 cells, it labeled NE‐enriched vesicles and real‐time imaged the NE exocytosis process triggered by high‐potassium stimulation; in brain tissue sections, dual‐immunofluorescence colocalization with DA *β*‐hydroxylase (DBH) antibody verified its specific labeling of noradrenergic neurons; and in vivo, it achieved fluorescence imaging of NE in mice. Notably, this study utilized the probe for the first time in a fluoxetine‐intervention rat model to directly visualize drug‐induced increases in brain NE levels, thereby providing a powerful real‐time imaging tool for investigating the mechanisms and evaluating the efficacy of antidepressants. These two studies advance the capability of in vivo NE detection from the perspectives of bioluminescence and fluorescence imaging, jointly expanding the technical boundaries of dynamic visualization in neurochemistry.

**FIGURE 3:**
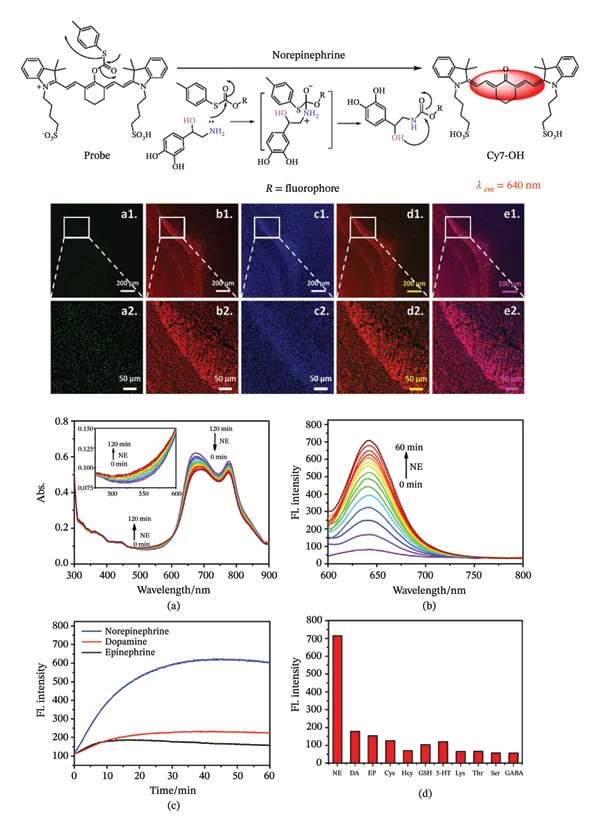
Sensing mechanism of probe 3 [26].

The SXU‐NEQ/F probe developed by the team of Yin Caixia and Huo Fangjun at Shanxi University, based on a “hunt‐and‐shoot” molecular mechanism, enables rapid and highly specific detection of NE [27]. Its dual‐site design shortens the response time to 12 min, and through optimization of steric hindrance and electronic effects, effectively avoids interference from molecules such as DA and cysteine (Cys). This probe was successfully used to visualize the dynamic release and reuptake of NE in PC12 cells, revealing the regulatory effects of *α*_2_ receptor drugs and the antidepressant venlafaxine on NE transport. After halogen modification, the probe can cross the blood–brain barrier, achieving for the first time real‐time imaging of the spatiotemporal distribution of NE in the brains of epileptic mice at the live‐animal level (Figure 4). The study showed that after epileptic seizures, whole‐brain NE levels are abnormally elevated, with significant accumulation in the thalamic region, while the loss of NE in the hippocampus is closely related to seizure‐associated GABAergic neuron damage. This work not only provides a high‐resolution tool for in vivo diagnosis of neuropsychiatric disorders but also opens new avenues for researching the neuroprotective mechanisms of NE in epilepsy pathology, offering important guidance for targeted drug development.

**FIGURE 4:**
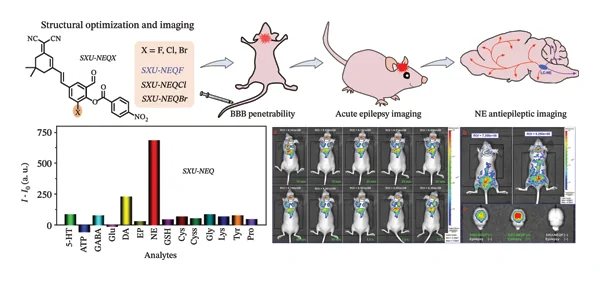
Sensing mechanism of probe SXU‐NEQ/F [27].

### 1.3. Millisecond Capture of Neural Dynamics

On the other hand, the BPS3 probe developed by the team of Tian Yang at East China Normal University innovatively employs a molecular folding‐water bridging dual‐acceleration mechanism, boosting NE detection speed to the 100 ms level [28]. This enables visualization of transient NE secretion at the neuronal plasma membrane (2‐s dynamic monitoring) and reveals the association between downregulated NE levels and antioxidant therapy in Alzheimer’s disease (AD) pathology (Figure 5). These two studies complement each other effectively; the former focuses on in vivo trans‐barrier detection and epilepsy pathology, while the latter breaks through the spatiotemporal resolution limit at the single‐cell level. Together, they advance precise observation of NE in neurodegenerative disease research. The ultrafast kinetic mechanism underlying BPS3 (conformational folding and water‐mediated energy barrier reduction) provides a new paradigm for biosensor design, and its capability for in vivo two‐photon imaging lays a technical foundation for early diagnosis and treatment evaluation of neuropsychiatric disorders.

**FIGURE 5:**
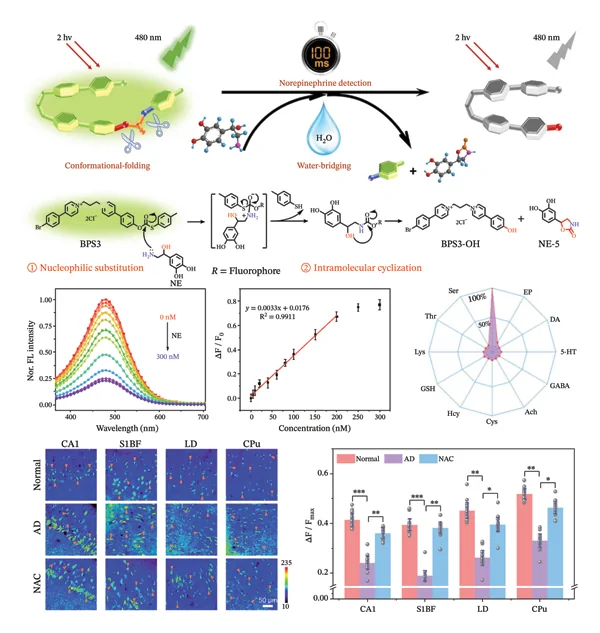
Sensing mechanism of probe BPS3 [28].

The resorufin‐based fluorescent probe LNE, developed by Gu et al., achieved significant breakthroughs targeting key bottlenecks in the real‐time dynamic monitoring of NE [29]. This study accomplished the first in situ visualization of NE in the brains of living depression models. By combining a chronic restraint stress model with open‐skull local perfusion technology, it was directly observed that the fluorescence intensity in specific brain regions of depressed mice was significantly weaker than that in the normal control group (*p* < 0.01), and successfully visualized the process of the antidepressant sertraline inhibiting NE reuptake. After 7 days of continuous sertraline treatment, the fluorescence signal in the brain regions showed a clear recovery (Figure 6). These findings not only provide direct in vivo imaging evidence supporting the “NE deficiency” hypothesis in depression pathology but also establish an imaging platform for the rapid evaluation of antidepressant efficacy. This work promotes the transformation of NE detection from in vitro analysis to in vivo pathological research. Its response time of less than 10 min and excellent brain region‐specific imaging capability set a new technological benchmark for mechanistic studies of neuropsychiatric disorders.

**FIGURE 6:**
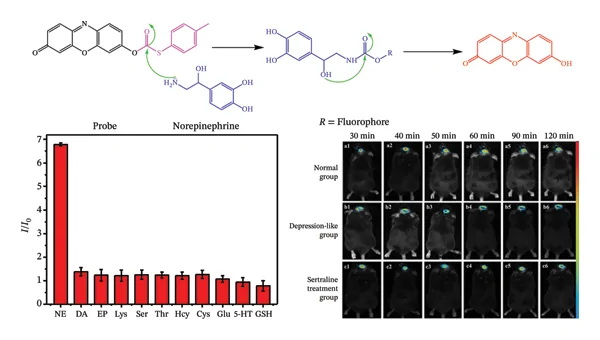
Sensing mechanism of probe LNE [29].

Tian Yang’s team constructed a supramolecular rationetric fluorescent sensor, CAN [30], utilizing the macrocyclic cavity of cucurbit [10] uril (Figure 7). This sensor integrates dual recognition sites comprising boronic acid‐functionalized naphthalimide and aldehyde‐modified coumarin. Upon specific binding with NE, a boronate ester condensation reaction is triggered, leading to a distinct ratiometric fluorescence change (F_435_/F_525_) with a response time of only 190 milliseconds. The team applied this sensor in freely moving mice, achieving real‐time monitoring of NE dynamics across 26 brain regions via an implanted optical fiber array. The study revealed that under fear stress, NE concentrations significantly increased to 4–5 μM in emotion‐related brain regions such as the basomedial amygdala (BMA), ventral posteromedial thalamic nucleus (VPM), and posterior lateral hypothalamus (PLH), whereas no significant change was observed in cortical areas. This research marks the first real‐time mapping of NE dynamics at a whole‐brain scale, clearly delineating the spatial gradient distribution of NE in key brain regions during emotional processing. It establishes a new paradigm for analyzing in vivo neurochemical circuits across multiple brain regions.

**FIGURE 7:**
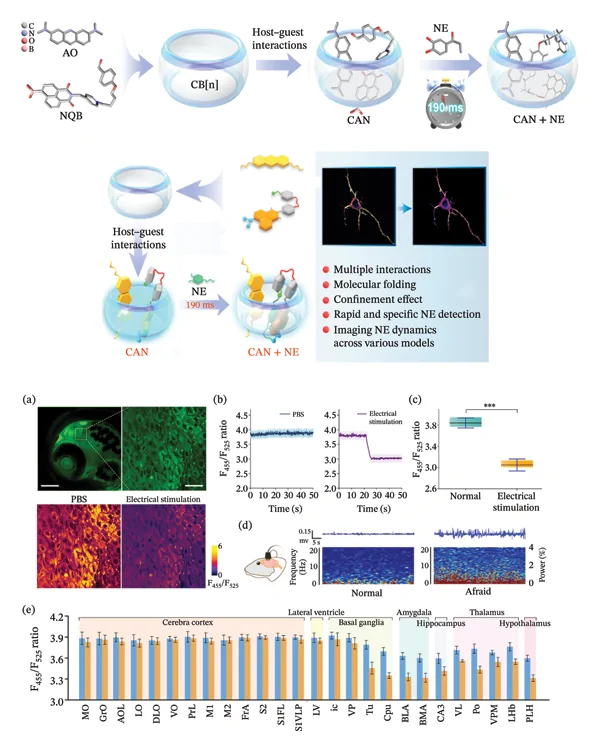
Sensing mechanism of probe CAN [30].

### 1.4. Precise Quantification: Anti‐Interference and Multitarget Synergistic Analysis

The precise detection of NE in the nervous system has long faced two core challenges, achieving specificity against structurally similar catecholamines (e.g., DA, EP), and suppressing background noise from high concentrations of endogenous interferents (e.g., amino acids, metal ions) in complex biological matrices like cerebrospinal fluid and plasma. Addressing these challenges requires moving beyond the traditional “lock‐and‐key” single‐site recognition toward a new generation of intelligent molecular recognition paradigms. Current technological breakthroughs are primarily embodied in three core concepts. First, the synergy of multiple recognition sites, which enables efficient spatial and energetic matching to NE’s characteristic functional groups (amine and catechol hydroxyls) through optimized design. Second, logic‐gated response strategies, which require the simultaneous presence of multiple specific biomarkers to trigger a signal, fundamentally avoid false positives caused by single‐factor interference. Third, multiparameter parallel resolution algorithms, which allow for the simultaneous discrimination of coexisting substances and signal decoupling. These innovations advance recognition precision to the level of molecular interactions, not only significantly improving the reliability of NE quantification in complex physiological environments but also establishing a methodological framework for “precisely extracting target signals from molecular noise.” This revolution in detection accuracy lays a solid technical foundation for deciphering the interaction mechanisms within neurotransmitter networks and the dynamic regulatory effects of microenvironmental parameters (e.g., viscosity, pH) on neurotransmitter release, propelling neurochemical research into a new era of precision.

Following this trend, the team led by Zhao Shulin at Guangxi Normal University successfully constructed a near‐infrared fluorescence‐photoacoustic dual‐modal probe, FPNE [31]. Upon reaction with NE, the probe releases the cyanine dye IR‐720, causing an absorption peak redshift from 585 nm to 720 nm and simultaneous enhancement of both fluorescence and photoacoustic signals at 750 nm. It shows a good linear response to NE in the range of 0.1–5 μM and exhibits over 15‐fold selectivity against structural analogs like DA and EP. At the in vivo level, NE in deep brain regions could be visualized in situ via multispectral optoacoustic tomography (MSOT) combined with fluorescence technology just 20 min after tail vein injection of FPNE (Figure 8). In vivo imaging comparisons revealed that the signal‐to‐background ratio (SBR) of MSOT was approximately twice that of fluorescence imaging. In a depressed mouse model application, NE signals recovered following intervention with the antidepressant fluoxetine (20 mg/kg), and the superior penetration and SBR advantage of photoacoustic imaging (about 2‐fold improvement over fluorescence) were further confirmed. This technology breaks through the spatiotemporal limitations of traditional detection methods, achieving for the first time cross‐scale dynamic monitoring of NE from synaptic vesicles (cellular level) to the whole brain. It provides an innovative visualization tool for parsing the pathological mechanisms of depression and evaluating personalized treatment.

**FIGURE 8:**
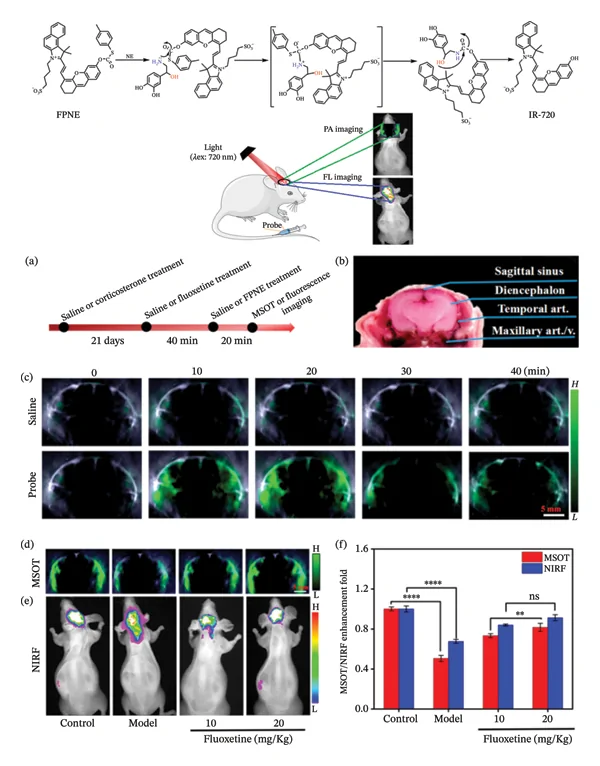
Sensing mechanism of probe FPNE [31].

The research team (Xinyi Xiong et al., Fujian Normal University) designed a dual‐channel near‐infrared fluorescent probe [32], RHO‐DCO‐NE. This probe ingeniously integrates two independent response mechanisms: a viscosity‐sensitive unit based on the intramolecular motion of a rotatable double bond within a rhodamine‐isophorone derivative (emission at 550 nm), and an NE‐specific recognition unit via a thioester group that reacts with NE (emission at 725 nm) (Figure 9). The spectral crosstalk between the two channels is negligible, ensuring reliable and simultaneous independent monitoring.

**FIGURE 9:**
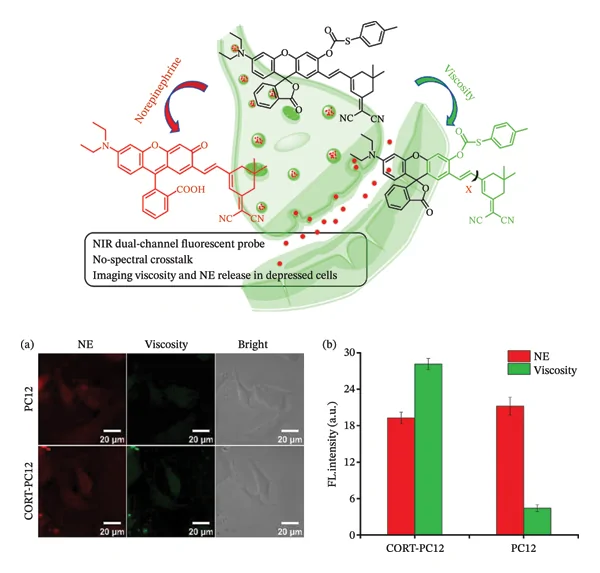
Sensing mechanism of probe RHO‐DCO‐NE [32].

Using this probe, the team uncovered, for the first time, the mechanism by which elevated cellular viscosity inhibits NE release in a cellular model of depression. Experiments showed that the intracellular viscosity in corticosterone‐induced depressive PC12 cells was significantly higher than in normal cells. When cellular viscosity was reduced by methods such as heating or adding the lysing agent Hemin, the NE efflux rate subsequently increased. This suggests that a high‐viscosity microenvironment may hinder the movement of neurotransmitter vesicles, leading to reduced NE release. This finding introduces a novel physicochemical perspective to depression pathology, implicating abnormal cellular viscosity as a previously underappreciated pathological factor.

This study not only provides a novel molecular tool capable of simultaneously and independently monitoring a specific neurotransmitter and its surrounding physical microenvironment but also proposes a new therapeutic approach for depression by modulating cellular viscosity to regulate neurotransmitter release. It thereby opens a new research direction for understanding the physical regulatory mechanisms underlying neurochemical transmission.

To address the challenge of low diagnostic specificity associated with single biomarkers in depression, Gao Yong’s team (Fujian Normal University) developed a dual‐responsive fluorescent probe [33], R‐NE‐hypochlorous acid (HClO), based on an “AND” logic gate. The core design of this probe integrates dual‐responsive moieties—a thiolactone and a thioester—to establish a cascade triggering mechanism: the fluorophore emitting at 533 nm is released only upon the simultaneous presence of both pathological molecules, NE and HClO, which sequentially induce spirolactone ring‐opening and thioester cleavage (Figure 10). In cellular validation, the probe clearly distinguished normal PC12 cells from corticosterone‐induced depression model cells and *β*‐amyloid‐induced AD model cells, with relative fluorescence signal intensity ratios of approximately 1:1.8:1.3. The underlying molecular basis for this discrimination lies in the distinct pathological signatures: depression model cells exhibit a “decreased NE release but elevated HClO levels” (reflecting enhanced oxidative stress), whereas AD model cells show a “low NE/high HClO” pattern. This “dual‐lock” design effectively eliminates interference from fluctuations in a single biomarker, significantly improving diagnostic specificity and offering a new approach for disease subtyping based on molecular signatures. Furthermore, experiments showed that the antioxidant N‐acetylcysteine could reduce HClO levels in model cells, providing preliminary experimental evidence for exploring antioxidant strategies in depression treatment.

**FIGURE 10:**
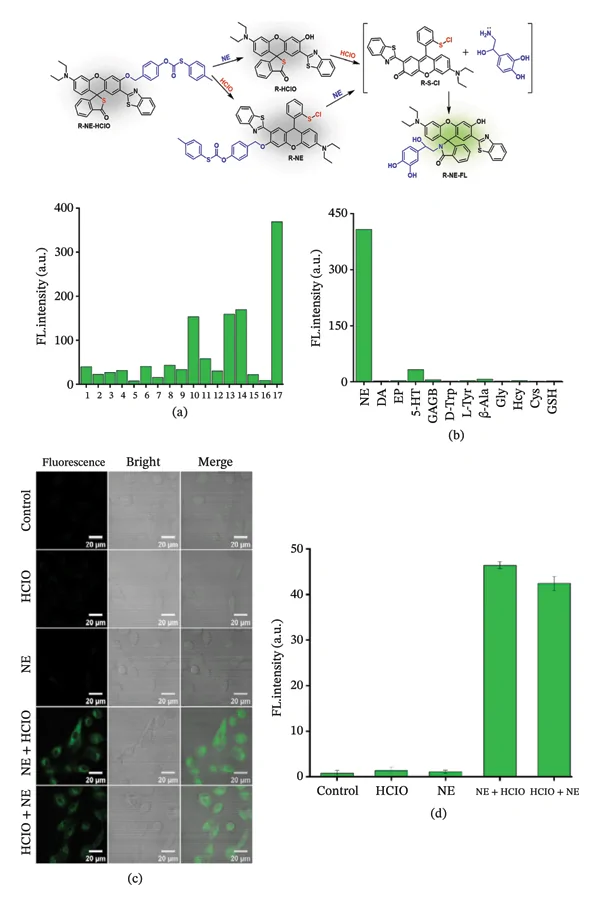
Sensing mechanism of probe R‐NE‐HClO [33].

### 1.5. High Specificity in Complex Matrices

The “dual‐lock” probe PBN‐5, developed by Qian Xuhong’s team at East China University of Science and Technology, employs an innovative aldehyde‐boronic acid‐silver ion ternary synergistic recognition strategy to significantly enhance the selectivity and reliability of NE detection within complex biological matrices [34]. This strategy integrates three key interactions: the aldehyde group condenses with the amine group of NE, the boronic acid specifically recognizes its catechol hydroxyl groups, and the silver ion forms a coordination bridge between the pyridyl nitrogen of the probe and the functional groups of NE (Figure 11). This multisite synergy increases the reaction rate by approximately 3.85‐fold, and the dual‐emission ratiometric signal output effectively minimizes interference from common endogenous substances like amino acids in plasma.

**FIGURE 11:**
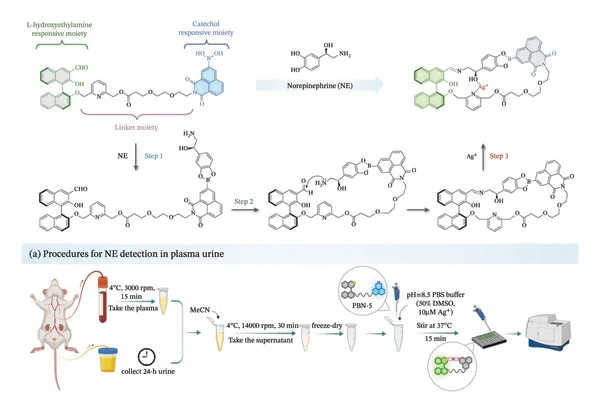
Sensing mechanism of probe PBN‐5 [34].

When applied in a spontaneously hypertensive rat model, the probe successfully quantified the differences in NE distribution under pathological conditions. The study revealed that the plasma NE concentration in model rats was as high as 31.51 nM, significantly elevated compared to the normal control group (15.36 nM). Concurrently, NE levels in kidney tissue were abnormally increased to 2.1 times that of the normal group. These findings provide direct and quantitative molecular imaging evidence supporting the classical hypothesis that sympathetic nervous overactivation is a key driver in the development and progression of hypertension.

### 1.6. Neurotransmitter Network Analysis

The pillar [5] arene‐based supramolecular probe CN‐DFP5, developed by the teams of Tian Yang and Sun Jing [35], offers an innovative solution for achieving high selectivity toward NE within environments containing multiple coexisting neurotransmitters. The probe features boronic acid‐functionalized naphthalimide and aldehyde‐modified coumarin recognition units attached to the upper and lower rims of the pillar [5] arene scaffold, respectively. Through synergistic host–guest interactions, it enables the parallel detection and discrimination of seven neurotransmitters, including NE (Figure 12). The core recognition mechanism involves a dual covalent capture of NE: the boronic acid group specifically binds its catechol hydroxyls to form a boronate ester, while the aldehyde group reacts with the amine to generate a Schiff base. The electron‐rich cavity of the pillar[5]arene further stabilizes the complex via electrostatic interactions. This mechanism endows NE with a unique dual‐channel fluorescence response pattern (enhancement at F_435_ and quenching at F_535_). Combined with principal component analysis (PCA), the response signal for NE can be clearly distinguished even from structurally similar DA and EP (PC1 contribution: 79.21%), with a cross‐validation accuracy of 94%. NMR and femtosecond transient absorption spectroscopy confirmed that NE binding inhibits the intramolecular charge transfer process of the probe (ICT/LE relaxation ratio decreased from 0.95 to 0.35), providing a mechanistic basis for the high selectivity.

**FIGURE 12:**
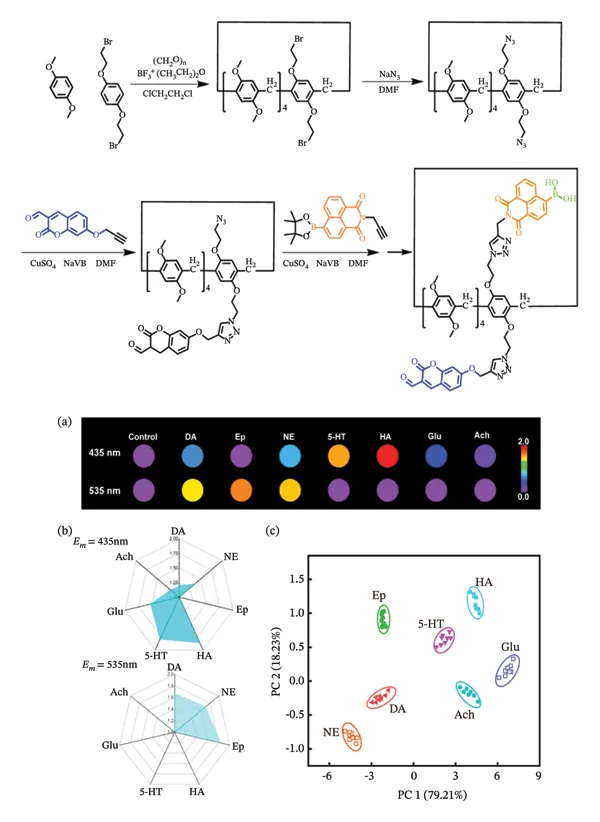
Sensing mechanism of probe CN‐DFP5 [35].

The probe also addresses the sensitivity and specificity bottlenecks for dynamic NE monitoring in living cells. Its excellent two‐photon properties (tissue penetration > 240 μm) and superior cell membrane permeability (colocalization coefficient with a membrane tracker: 0.97) enable efficient neuronal labeling. Upon exogenous NE stimulation, characteristic dual‐channel fluorescence changes were observed in the cytoplasm. By establishing a concentration‐fluorescence response linear model (limit of detection: 1 μM, linear range: 1–21 μM) and employing PCA to process the dual‐channel signals, this study achieved, for the first time, real‐time discrimination of NE from six other coexisting neurotransmitters in living cells. This provides a tool for simultaneous multitarget monitoring to study chemical coding in neuronal communication.

CN‐DFP5 demonstrates remarkable anti‐interference capability in complex biological samples. In simulated cerebrospinal fluid containing high concentrations of amino acids, reactive oxygen species, or metal ions, the recovery rate for NE remained above 95%. In practical application within an AD mouse model, the probe successfully achieved accurate quantification of exogenously added NE in cerebrospinal fluid (error < 5%) and clearly visualized the spatial distribution of NE in the hippocampal region via three‐dimensional two‐photon imaging. This “single‐probe‐multitarget” strategy breaks through the technical limitations of traditional methods that rely on multiplexed labeling, offering a new paradigm for deciphering the complex multineurotransmitter pathology in neurodegenerative diseases.

### 1.7. Functional Expansion: From Detection to Integrated Diagnosis and Therapy

The pathogenesis of neuropsychiatric disorders is closely linked to the complex dysregulation of neurotransmitter homeostasis. The conventional separation of diagnosis and treatment in research has significantly hindered the realization of precision medicine. Against this backdrop, the emergence of theranostic probe technology is driving a fundamental paradigm shift in the field. Its core design philosophy lies in integrating diagnostic and therapeutic modules into a single system through intelligent molecular engineering that responds to the pathological microenvironment.

Specifically, this paradigm encompasses two tightly coupled dimensions: in the diagnostic dimension, the probe can real‐time perception disease‐specific biomarkers (such as abnormally elevated oxidative stress molecules or enzyme activity) and convert them into in situ, quantifiable optical or signal readouts, thereby enabling dynamic visual monitoring of the pathological process; in the therapeutic dimension, the diagnostic signal can further trigger the controlled release system of a built‐in drug, precisely regulating NE levels or repairing related pathways at specific spatiotemporal scales to achieve targeted intervention.

This closed‐loop operation of “sensing–feedback–intervention” breaks through the passive nature of traditional tools and achieves three key leaps: First, it shifts disease diagnosis from static endpoint assessment to continuous process monitoring, allowing for the dynamic capture of pathological progression trajectories. Second, it significantly enhances the spatiotemporal precision and biosafety of treatment through microenvironment‐driven prodrug activation strategies. Third, it establishes a real‐time feedback mechanism for therapeutic efficacy, enabling the dynamic optimization of individualized treatment plans.

In summary, the theranostic paradigm not only provides a novel perspective for understanding the spatiotemporal dynamics of neurotransmitter imbalance but also marks a strategic transformation in neurological disease research from “describing phenomena” to “active regulation,” laying a crucial foundational technical framework for the future of precision neuromedicine.

### 1.8. Simultaneous Diagnosis and Therapy

Tang Bo’s team (J. Am. Chem. Soc. 2025) developed a theranostic fluorescent probe, Cou‐NE‐H_2_O_2_ [36], which integrates the dynamic regulation of NE with the visualization of oxidative stress, aiming to overcome a key bottleneck in the clinical translation of depression therapy (Figure 13). The probe consists of a coumarin fluorophore, a hydrogen peroxide (H_2_O_2_)‐responsive phenylboronate ester unit, and an NE prodrug module. Under conditions of abnormal reactive oxygen species levels in the brains of depressed mice, Cou‐NE‐H_2_O_2_ responds to excessive H_2_O_2_, converting the phenylboronate ester group into a phenol structure; subsequent electron rearrangement triggers the cleavage of the carbonate bond, simultaneously releasing NE and the coumarin fluorophore, which generates a fluorescent signal.

**FIGURE 13:**
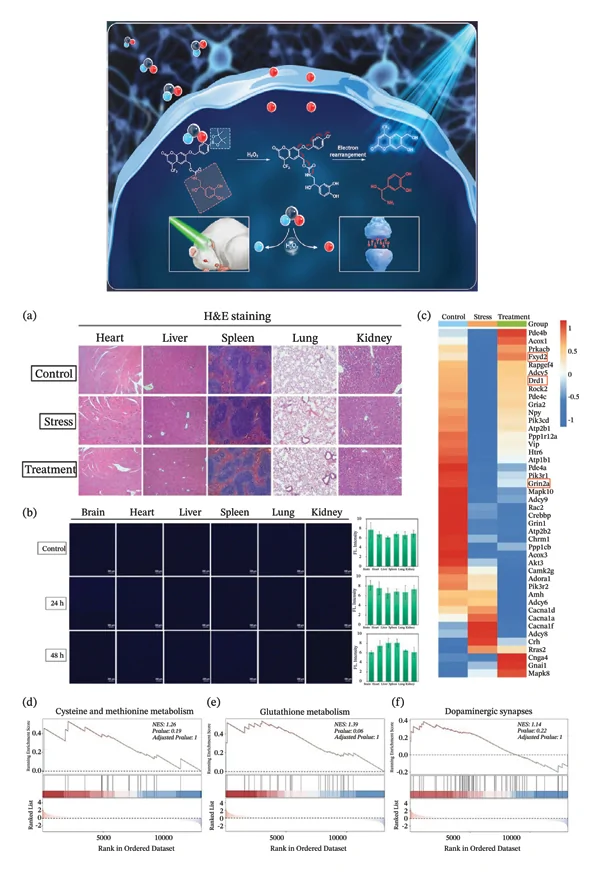
Sensing mechanism of probe Cou‐NE‐H_2_O_2_ [36].

On the diagnostic front, the probe efficiently crosses the blood–brain barrier and specifically reacts with abnormally overexpressed H_2_O_2_ in the brains of depressed model mice (levels approximately 2.1 times higher than in the normal group), releasing the coumarin fluorophore that emits at 460 nm. The fluorescence intensity shows a linear relationship with H_2_O_2_ concentration in the range of 0–50 μM, enabling clear discrimination of oxidative stress levels between brain tissues of depressed and normal mice (fluorescence intensity difference *p* < 0.01). This provides an objective imaging biomarker for depression based on oxidative stress.

On the therapeutic front, the H_2_O_2_‐triggered NE release efficiency reaches 69%. After 2 weeks of treatment, the brain NE concentration in depressed model mice recovered to normal levels (verified by ELISA), and the expression of brain‐derived neurotrophic factor (BDNF) was also upregulated to the physiological range. Behavioral tests showed that a probe dose of only 0.34 mg/kg significantly ameliorated anhedonia (sucrose preference increased from 45% to 85%) and reduced despair behavior (immobility time in the forced swim test decreased by 60%). The onset of action of the probe (approximately 2 weeks) was superior to that of traditional antidepressants (typically 4 weeks). Further transcriptomic analysis revealed that probe treatment upregulated the expression of cAMP pathway‐related genes such as Grin2a and Drd1, enhanced glutathione metabolism, and improved neurosynaptic plasticity (KEGG enrichment analysis *p* < 0.001). These findings elucidate, at the molecular level, a novel antidepressant mechanism involving the synergistic regulation of NE and oxidative stress.

The near‐infrared hemicyanine probe NIR‐NE, developed by the teams of Zhou Na and Yin Caixia, has provided crucial new insights into the NE‐related pathology of depression [37]. This study suggests that the core issue in depression may lie in a dynamic dysfunction of NE secretion, rather than a simple deficiency in its static content. In a corticosterone‐induced PC12 cell model of depression, quantitative flow cytometry analysis revealed no significant difference in the total NE content between depressed and normal cells (mean fluorescence intensity, MFI: 102.3 ± 8.7 vs. 98.6 ± 7.2). However, real‐time monitoring of high‐potassium (50 mM K^+^)‐induced exocytosis using the probe uncovered a key pathokinetic feature: the NE secretion rate in depressed cells (fluorescence decay slope: 0.11 min^−1^) was only about 34% of that in normal cells (0.32 min^−1^) (Figure 14). This finding offers an important refinement to the traditional “NE deficiency hypothesis” from the perspective of secretory dynamics.

**FIGURE 14:**
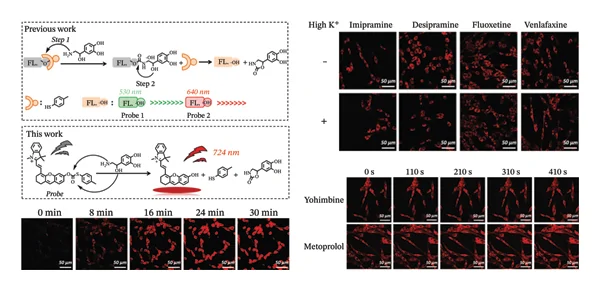
Sensing mechanism of probe NIR‐NE [37].

Further mechanistic investigations showed that the *α*_2_‐adrenergic receptor antagonist yohimbine significantly inhibited NE secretion in normal cells (slope reduced to 0.15 min^−1^), while the NE reuptake inhibitor desipramine restored the secretory function in depressed cells to near‐physiological levels (slope increased to 0.28 min^−1^). These results collectively demonstrate that dysregulation of the G protein‐coupled receptor (GPCR)–mediated secretory pathway is a primary cause of the NE secretory dysfunction. Moreover, with its excellent deep‐tissue penetration (> 150 μm) and resistance to autofluorescence interference, this near‐infrared probe establishes a new technological paradigm for in vivo studies of real‐time neurotransmitter secretion kinetics.

## 2. Conclusion

This review summarizes the organic small‐molecule fluorescent probes for NE, highlighting several common characteristics across three aspects: design strategy, performance optimization, and biological applications.

At the molecular recognition level, to achieve precise differentiation within the catecholamine family, researchers have primarily constructed recognition sites around the unique *β*‐hydroxyethylamino structure of NE, enabling specific detection through nucleophilic substitution–cyclization cascade reactions. In addition to covalent reaction pathways, recognition mechanisms based on supramolecular interactions such as host–guest inclusion and hydrogen bonding have also been extensively explored. Together, these approaches constitute a diversified strategy system that effectively enhances the selective recognition capability of probes for NE.

At the response performance level, researchers have made continuous breakthroughs in kinetic response speed and signal output modes. Through strategies such as modulating reaction transition states, water molecule catalysis, and molecular conformational optimization, the response time of probes has been significantly shortened from the minute scale to hundreds of milliseconds or even 60 milliseconds, enabling instantaneous capture of neurotransmitter release processes. Meanwhile, the introduction of signal optimization strategies such as ratiometric response and near‐infrared emission has markedly improved the quantitative accuracy and tissue penetration depth for in vivo imaging.

At the bioimaging application level, current probes have been successfully applied to the study of pathological mechanisms in neurodegenerative diseases such as AD, Parkinson’s disease, and depression at both cellular and in vivo levels, showing preliminary potential for theranostic integration. Furthermore, research into the comorbidity mechanisms between NE and physiological diseases such as hypertension has gained new insights and directions through the use of these visualization tools.

The innovation of NE detection technology is driving neuroscience research from static description into a new stage of dynamic visualization and precise modulation. This review highlights progress in molecular probe design that achieves spatial mapping from organelle microenvironments to whole‐brain scales, alongside temporal resolution at the millisecond level—enabling real‐time tracking of transient NE release, diffusion, and reuptake. Through strategies such as multirecognition site synergy, logic‐gated responses, and multiparameter parallel analysis, the selectivity and anti‐interference capability of probes within complex biological systems have been significantly enhanced. Furthermore, the emergence of integrated diagnostic and therapeutic probes enables real‐time sensing of the pathological microenvironment and active regulation of NE levels, providing a new paradigm for precise intervention in neuropsychiatric disorders.

Despite these advances, several challenges persist. The biocompatibility and long‐term stability of probes, quantitative signal calibration, and clinical translation remain critical issues to be resolved. Future directions with significant potential include multimodal integrated probes, wireless real‐time monitoring systems, and artificial intelligence‐assisted data analysis. Future design of fluorescent probes for neurotransmitters should focus on three synergistic directions: First, constructing a modular design platform that enables specific recognition of different neurotransmitters by simply replacing the recognition group, thereby improving research and development efficiency. On this basis, we aim to develop multiplexed synchronous imaging techniques for simultaneous monitoring of dynamic changes in multiple neurotransmitters within the same biological system. Finally, greater emphasis should be placed on clinical translation to ensure that probes feature favorable long‐term biostability, low toxicity, and blood–brain barrier permeability, thereby advancing their translation from animal models to practical clinical applications..

## Funding

No funding was received for this manuscript.

## Conflicts of Interest

The authors declare no conflicts of interest.

## Data Availability

Research data are not shared.
